# Onychomycosis in Northwestern Greece over a 7-Year Period

**DOI:** 10.3390/pathogens9100851

**Published:** 2020-10-17

**Authors:** Hercules Sakkas, Christos Kittas, Georgia Kapnisi, Efthalia Priavali, Amalia Kallinteri, Ioannis D. Bassukas, Konstantina Gartzonika

**Affiliations:** 1 Microbiology Department, Faculty of Medicine, School of Health Sciences, University of Ioannina, 45110 Ioannina, Greece; 2 Department of Microbiology, University Hospital of Ioannina, 45110 Ioannina, Greece; md03580@uoi.gr (C.K.); g.kapnisi@uoi.gr (G.K.); epriaval@cc.uoi.gr (E.P.); pmd0080@uoi.gr (A.K.); 3 Department of Skin and Venereal Diseases, Faculty of Medicine, School of Health Sciences, University of Ioannina, 45110 Ioannina, Greece; ibassuka@uoi.gr

**Keywords:** onychomycosis, dermatophytes, yeasts, molds, *Candida* spp., *Trichophyton* spp., Greece

## Abstract

Onychomycosis is considered as one of the major public health problems with a global distribution associated with geographic, demographic and environmental factors, underlying comorbidities and immunodeficiency disorders. This study was conducted to investigate the etiological agents of onychomycosis, in Northwestern Greece during a 7-year period. The study population included 1095 outpatients with clinically suspected onychomycosis that presented to the University Hospital of Ioannina, NW Greece (2011–2017). Samples were examined for causative fungi, and mycological identification was established using standard mycological methods. Demographic data of each patient, comorbidities, localization of infection and history of previous fungal infection were collected. Onychomycosis was diagnosed in 317 of the 1095 suspected cases (28.9%) and the most frequently isolated pathogens were yeasts (50.8%) followed by dermatophytes (36.9%) and non-dermatophyte molds (NDMs) (12.3%). Dermatophytes were mostly involved in toenail onychomycosis (90.6%) and more commonly affected males than females (57.3% vs. 42.7%), while the predominantly isolated pathogen was *Τrichophyton rubrum* (74.4%) followed by *Τrichophyton interdigitale* (21.4%). *Candida albicans* was the most prevalent isolated yeast (82%), whereas among the cases with onychomycosis due to NDMs, *Aspergillus* spp. were isolated as the principal species (59%). Continuous monitoring should be performed in order to identify possible trends and shifts in species isolation rates and to evaluate the impact of onychomycosis among the general population and high-risk groups.

## 1. Introduction

Onychomycosis is a common fungal infection of the nails, which is responsible for up to 50% of all nail abnormalities, usually caused by dermatophytes, non-dermatophyte molds (NDMs) and yeasts [1]. The infection is more common in toenails than fingernails and among dermatophytoses the most common causative agent is the anthropophilic species *Trichophyton rubrum* [2]. An effective treatment approach is rather difficult, especially in toenail onychomycosis, which is considered one of the most frequently encountered superficial mycoses worldwide affecting both the patient’s health and quality of life [3].

Onychomycosis is characterized clinically by disorganized, fragile, thick and discolored nails. Four different clinical patterns of onychomycosis are distinguished according to the direction of the invasion of the pathogen relative to the anatomy of the nail apparatus: distal subungual, (the most common type), proximal subungual, superficial, and total dystrophic onychomycosis [4]. The infection can be complicated by cellulitis, osteomyelitis and soft tissue and bone necroses in diabetic patients and immunocompromised individuals. Severe extensive toenail involvement and subungual hyperkeratosis have been described in HIV-infected individuals [5]. Insomuch that accuracy of clinical diagnosis alone is often limited, laboratory diagnostic procedures including direct microscopic examination and mycological culture can detect and discriminate causative fungi in clinical specimens. In addition, several molecular assays based on polymerase chain reaction have been developed and evaluated, since mycological identification and species’ determination is rather difficult to obtain by microscopy [6]. Optical techniques have also been reported as promising methods for the evaluation of onychomycosis in modern clinical diagnostics including optical coherence tomography (OCT), confocal laser scanning microscopy (CLSM), and Raman spectroscopy (RS) [7,8].

The constantly increasing prevalence of onychomycosis has been reported to be as high as 10% within the general population and up to 50% in elderly individuals, more frequently occurring in males than in females [2]. According to a recent systemic review, the mean prevalence rate of the disease in the general population was 11.4% (5.0–17.7%) [9]. Differences in the prevalence and the etiological agents of onychomycosis may exist depending on several geographic, environmental and demographic predisposing factors. Risk factors such as advanced age, male gender, occupation, obesity, smoking, and underlying comorbidities including diabetes mellitus, nail trauma, psoriasis, peripheral vascular disease, acquired immunodeficiency syndrome and other congenital or acquired immunodeficiency disorders are also associated with high infection rates [1,2,10].

However, the prevalence of the disorder may vary significantly even between different regions within the same country and the knowledge of the locally predominant causative agents in each region may contribute to a rational approach to the treatment, a framework for the interpretation and better understanding of related risk factors, and as reference for future research. The aim of the present study was to report the distribution of the causative agents isolated from onychomycosis specimens over a period of seven years (2011–2017) in a tertiary hospital based in Northwestern Greece.

## 2. Results

**Figure 1:**
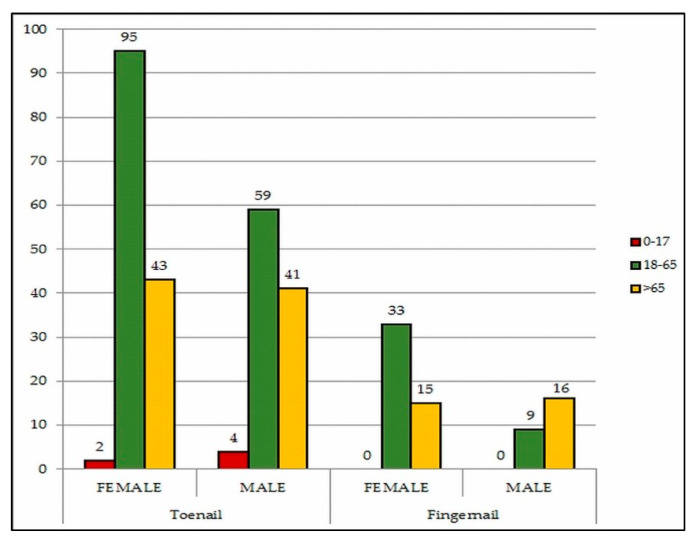
Distribution of onychomycosis according to age group, gender and localization.

Of the 1095 suspected cases, onychomycosis was confirmed in 317 (28.9%) patients, ranging in age from 3 to 86 years (58 ± 17.1). The fungal isolation rate increased with age, and 115 out of 317 patients (36.3%) were aged over 65 years. Female study population outnumbered male by index 1.7 and prevalence of onychomycosis in females (59.3%) was higher than in males (40.7%). However, a slightly higher infection rate was seen in males (31.9%) compared to females (27.2%). Toenail onychomycosis was present in 80.6% of total positive male cases and compared to fingernail was more frequent in total (Figure 1).

**Figure 2:**
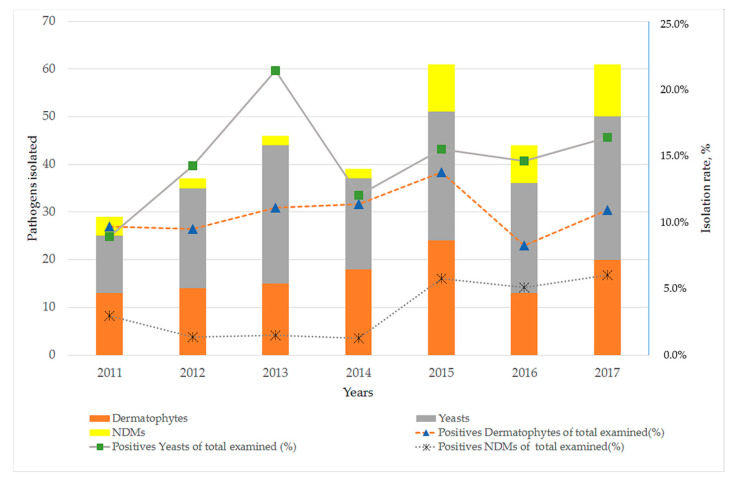
Annual distribution of fungi recovered from 2011 to 2017 according to the causative agent.

According to past medical history 87 out of 317 patients (27.4%) had fungal skin or/and nail infection or nail trauma. Furthermore, associated comorbidities were reported in 120 out of 317 diagnosed cases (37.9%); diabetes mellitus was the predominant abnormality (18.3%), followed by immunosuppression (5.4%), rheumatoid arthritis (3.5%) and psoriasis (3.2%). Regarding the main etiological agent involved in onychomycosis, the most frequently isolated pathogens in our study were yeasts (50.8%), followed by dermatophytes (36.9%) and NDMs (12.3%). Annual distribution of the investigated individuals and isolation rate of fungi recovered from 2011 to 2017 is shown in Figure 2.

Analysis in respect to gender revealed that dermatophytes and yeasts were the predominant pathogens isolated in men (detected in 67 out of 129 positive male cases, 51.9%) and women (detected in 115 out of 188 positive female cases, 61.2%), respectively. Dermatophytes were more involved in toenail onychomycosis (90.6%) than in fingernail (9.4%). The most encountered pathogen was *Τ. rubrum* (74.4%) followed by *Τrichophyton interdigitale* (21.4%). Among yeasts, *Candida albicans* was the most prevalent isolated species (82%). Yeasts were the major causative agents involved in fingernail onychomycosis especially in the female population (83%). In the 39 cases with onychomycosis due to NDMs, *Aspergillus* spp. were isolated as the principal species (59%), followed by *Scopulariopsis brevicaulis* (20.5%) (Table 1).

## 3. Discussion

Onychomycosis constitutes the most frequent nail disorder with a global distribution associated with geographic, demographic and environmental factors, and a significant spectrum of comorbidities [1,2,10]. During recent decades, prevalence rates of onychomycosis have risen due to variable socio-economic factors, social and family trends and cultural diversity. With a modern lifestyle, the widespread use of antibiotics and anti-inflammatory drugs may potentially predispose to the infection [4,11]. In addition, variation may underline the distinct differences in the incidence of fungal nail infections across different countries, but also between different regions within the same country [12].

However, specific information on the risk factors of onychomycosis pathogenesis among different population groups is scarce. Incidence rates in different studies vary widely from 9.1% to 13.8% in North American [13,14], from 14.2% to 50.8% in European [15,16,17,18,19,20] and even from 8.8% to 78.6% in African countries [21,22,23,24]. Toenail onychomycosis and dermatophytes are commonly found in temperate western countries, while Candida species, NDMs and fingernail onychomycosis are more frequently involved in countries with a hot and humid climate [10]. Ioannina, located in Northwestern part of Greece, is characterized by mild climate, generally warm to temperate but also with high humidity which might predispose to fungal infection [25]. This is in accordance with the predominance of yeasts and with the higher infection rate of onychomycosis in Northwestern Greece, when comparing our results with the findings of other studies from Greece. Thus, the isolation rate of onychomycosis among the suspected cases in this study was 28.9%, while in two studies from Crete and one from Southern Greece, two regions with lower relative humidity rates on an annual basis, the corresponding rates were 24.3% [25], 16.5% [26] and 15.7% [12], respectively.

Dermatophytes are the most important cause of fungal nail infections, including *T. rubrum* and *T. interdigitale*, which are the most predominantly isolated species in about 80–90% of cases [2]. Less commonly, *Epidermophyton floccosum, Trichophyton violaceum, Microsporum gypseum, Trichophyton tonsurans, Trichophyton soudanense* and *Trichophyton verrucosum* have also been identified as causative agents of onychomycosis [27]. Furthermore, the incidence of onychomycosis due to geographical distribution of dermatophytes from their endemic environments to new geographically remote areas has increased during recent years because of mass population mobility as a result of migration, troop movements and recreational travel [2,27]. In our study, *T. rubrum* was the most frequently isolated species among dermatophytes, followed by *T. interdigitale*, both involved predominantly in toenail infections, while only five fungal isolates (4.3%) belonging to three species other than the aforementioned pathogens were isolated. The results of our study demonstrated that dermatophytes mostly affected toenails and this was found to be in agreement with similar findings from other European countries [17,18,19,20].

On the other hand, yeasts such as *C. albicans* and *Candida parapsilosis* and NDMs of the genera *Scytalidium, Scopulariopsis* and *Aspergillus* are also associated with nail infections as primary or opportunistic pathogens [27]. Although yeasts are widespread in tropical and subtropical regions, the status of candidiasis as a primary causative agent of onychomycosis in our report is consistent with other studies that have been conducted in Greece [12], Italy [15] and Spain [16]. Regarding *Candida* species, *C. albicans* was the most common causative agent of fingernail onychomycosis, especially in female individuals. As discussed in previous reports [25,26], this could be attributed to females’ predominant occupation in wet workplaces and household environments [25,26]. This is a finding like that from other studies undertaken in Greece [12,25,26], Italy [18] and Serbia [20]. In contrast, surveys from Israel [28] and Turkey [29] identified *C. parapsilosis* and *C. tropicalis* as the most frequently isolated yeasts, respectively. Moreover, during the last three years the isolation rate of NDMs seems to have progressively increased (Figure 2) but is still relatively low in comparison to corresponding data in the literature [1,2,30]. Remarkably, in a study undertaken in Malaysia, NDMs were isolated in 69.3% of onychomycosis cases [31].

There is also a substantial impact of several risk factors and comorbidities underlying the pathogenesis and distribution of onychomycosis. Advanced age is an important risk factor in onychomycosis’ development and according to our results more than one-third of the infected individuals were over 65 years old, while children aged 0-17 were less infected (1.9%). Both the most increased and the lowest fungal isolation rates of onychomycosis among elderly patients and children, respectively, are consistent with the results observed in other studies [13,17,20,32]. Age-associated immune deficits in association with several disorders, consecutive nail trauma, or dystrophic and thickened nails may predispose to onychomycoses among elderly patients [4,5,20].

Among underlying disorders, diabetes mellitus was the most predominant causative agent in our study. It has been well documented that onychomycosis is frequently observed in at least one-third of diabetic patients, commonly associated with tinea pedis, and the prevalence of onychomycosis is almost threefold higher in these patients than in non-diabetic individuals [1,2,5,10,17]. According to our results, a small proportion of diagnosed cases (3.2%) were also patients with psoriasis. It has been reported that psoriasis patients are more likely to develop onychomycosis than the general population [8,33,34]. Nevertheless, considering that both onychomycosis and nail psoriasis usually present with similar clinical features leading to difficult discrimination and ambiguous results, some reports indicate no differences in prevalence rates between psoriasis and non-psoriasis patients with clinical nail abnormalities [35,36].

## 4. Materials and Methods

The study population included outpatients with clinically suspected onychomycosis who presented to the University Hospital of Ioannina located in the Northwestern part of Greece. During a 7-year period (2011–2017) samples from 1095 patients were examined at the Microbiology Laboratory for causative fungi. Demographic data of each patient such as age, gender, urban or rural residency and occupation, comorbidities (diabetes, psoriasis, rheumatoid arthritis, immunosuppression), localization of infection on toenails, fingernails or on both and history of previous onychomycosis or skin fungal infection in other sides were collected. Excluded patients were those on systemic antifungal therapy during the last month or on topical therapy during the last fifteen days.

The nail surface was firstly cleaned with 70% ethanol, and samples were taken by scraping or clipping the affected areas. Nail scrapings/clippings were collected in sterile Petri dishes and divided in three portions. Mycological identification was established using standard mycological methods. A portion of samples was subjected to direct microscopy at ×100 and ×400 magnifications after the addition of 20% KOH. Another part of the materials was inoculated on Sabouraud CAF Agar (Liofilchem, Roseto degli Abruzzi, Italy), Sabouraud Chloramphenicol Actidion Agar (BioLab Diagnostics, Budapest, Hungary) and/or on Dermatophyte Agar tubes (bioMerieux, Marcy L’Etoile, France). The incubation of inoculated media was performed at 25–27 °C and cultures were checked out twice weekly for 28 days before being declared as negative. The remaining samples were stored and plated again if fungi failed to grow.

The results were based on the interpretation of each mycological procedure: (i) The microscopic findings were considered positive if fungal elements (arthroconidia, blastoconidia, true hyphae or pseudo-hyphae) were present in the preparation. (ii) Cultures yielding dermatophytes were considered positive. In cases of NDMs or yeast identification, positivity of cultures was attributed when the same single organism grew on repeated cultured samples obtained from the same untreated patient. Additionally, a positive result from microscopy was mandatory for a culture yielding NDMs or yeasts. Dermatophytes and NDMs were identified by macroscopic and microscopic characteristics while yeasts were identified by morphology, growth after re-cultivation on CHROMID^®^ Candida agar (bioMerieux, Marcy L’Etoile, France) and by using API 20C AUX test (bioMerieux, Marcy L’Etoile, France).

The study was conducted in accordance with the Declaration of Helsinki, and the protocol was approved by the Scientific Committee of the University Hospital of Ioannina (Approval Document: 696/01-09-2020, Project identification code: 15/31-08-2020, s.11).

## 5. Conclusions

In conclusion, in Northwestern Greece onychomycosis infections occur mainly in the elderly, involve toenails more frequently than fingernails and result from *C. albicans* and *T. rubrum* that are predominantly isolated from material sent for the microbiological evaluation of onychomycosis. Future studies, planned to co-evaluate variable epidemiological, clinical and environmental data, are needed in order to better interpret this finding. Moreover, long-term, prospective monitoring is imperative in order to delineate the reasons for possible trends and shifts in species isolation rates as well as to recognize the mechanisms underlining the emergence of new species as pathogens of onychomycosis in a certain population.

## Figures and Tables

**Table 1:** Frequency and causative agents of onychomycosis due to dermatophytes, yeasts and non-dermatophyte molds (NDMs).

Fungi Groups	Total	Total (%)	Toenail	Toenail (%)	Fingernail	Fingernail (%)
Dermatophytes	117	36.9%	106	90.6%	11	9.4%
*T.rubrum*	87	27.4%	78	73.6%	9	81.8%
*T.interdigitale*	25	7.9%	24	22.6%	1	9.1%
*T.tonsurans*	2	0.6%	1	0.9%	1	9.1%
*T.violaceum*	1	0.3%	1	0.9%	0	0.0%
*E.floccosum*	2	0.6%	2	1.9%	0	0.0%
Yeasts	161	50.8%	105	65.2%	56	34.8%
*Candida albicans*	132	41.6%	83	79.0%	49	87.5%
*Candida non-albicans*	14	4.4%	11	10.5%	3	5.4%
*Rhodotorula* spp.	15	4.7%	11	10.5%	4	7.1%
Molds	39	12.3%	33	84.6%	6	15.4%
*Penicillium* spp.	3	0.9%	2	6.1%	1	16.7%
*Scopulariopsis brevicaulis*	8	2.5%	8	24.2%	0	0.0%
*Cladosporium* spp.	1	0.3%	1	3.0%	0	0.0%
*Fusarium* spp.	2	0.6%	1	3.0%	1	16.7%
*Aspergillus* spp.	8	2.5%	6	18.2%	2	33.3%
*Aspergillus niger*	6	1.9%	5	15.2%	1	16.7%
*Aspergillus terreus*	9	2.8%	8	24.2%	1	16.7%
*Acremonium* spp.	2	0.6%	2	6.1%	0	0.0%
Total	317	100.0%	244	77.0%	73	23.0%
